# Serum Cytokine Signature That Discriminates *Helicobacter pylori* Positive and Negative Juvenile Gastroduodenitis

**DOI:** 10.3389/fmicb.2016.01916

**Published:** 2016-12-15

**Authors:** Svetlana F. Khaiboullina, Sayar Abdulkhakov, Alsu Khalikova, Dilyara Safina, Ekaterina V. Martynova, Yuriy Davidyuk, Felix Khuzin, Rezeda Faizullina, Vincent C. Lombardi, Georgi V. Cherepnev, Albert A. Rizvanov

**Affiliations:** ^1^Institute of Fundamental Medicine and Biology, Kazan Federal UniversityKazan, Russia; ^2^Kazan State Medical UniversityKazan, Russia; ^3^Nevada Center for Biomedical ResearchReno, NV, USA; ^4^Department of Clinical Laboratory Diagnostics, Kazan State Medical AcademyKazan, Russia

**Keywords:** *H. pylori*, virulence factors, cytokines, gastroduodenitis

## Abstract

Gastroduodenitis caused by *H. pylori*, often acquired in early childhood, is found in about 50% of the adult population. Although *H. pylori* infections can remain asymptomatic, its virulence factors usually trigger epithelial vacuolization and degeneration, loss of microvilli, disintegration of cytoplasm, and leukocyte accumulation. It is believed that leukocyte infiltration is driven by cytokines produced locally in infected tissue. However, so far little is known about changes in serum cytokines in juvenile patients infected with *H. pylori*. Serum cytokine profiles were analyzed in 62 juvenile patients diagnosed with gastroduodenitis using the Bio-Plex multiplex assay. *H. pylori* infection was confirmed in 32 patients, while 30 patients were *H. pylori*-free. Cytokines CXCL5 and CXCL6, potent neutrophil chemoattractants, were upregulated in all patients diagnosed with gastroduodenitis. Serum levels of IL8, a prototype neutrophil attractant, remained unchanged in subjects with gastroduodenitis relative to controls. Therefore, our data suggest that CXCL5 and CXCL6 play a role in directing neutrophil trafficking into inflamed gastroduodenal tissue. In addition, the CCL25/GM-CSF ratio differed significantly between *H. pylori*-positive and -negative juveniles. Further, study is needed to evaluate the role of CCL25 and GM-CSF in the pathogenesis of the different etiologies of gastroduodenitis.

## Introduction

*Helicobacter pylori* (*H. pylori*) was identified in 1982 and suggested to be a causative agent for gastritis and stomach ulcers (Marshall and Warren, 1984). This helix-shaped gram-negative bacterium colonizes gastric mucosa and persists as a chronic infection (Marshall et al., 1985a; Morris and Nicholson, 1987). *H. pylori* is one of the most common gastrointestinal infections, being found in about 50% of the adult population (Sipponen et al., 1996; Kosunen et al., 1997). The majority of *H. pylori* infections remain asymptomatic, with only 15% of carriers developing symptoms (Atherton, 1998; Ernst and Gold, 2000). Infection, often acquired in early childhood, has been shown to be associated with poor hygiene and impoverished living conditions (Malaty and Graham, 1994; Kivi et al., 2003; Konno et al., 2005; Dattoli et al., 2010). It is believed that *H. pylori* is transmitted via fecal-oral or oral-oral routes (Goh et al., 2011).

During gastric epithelium colonization, *H. pylori* establishes a persistent infection within the mucus layer without crossing the epithelial barrier (Noach et al., 1994). Infection is histologically characterized by surface epithelial degeneration, inflammation, and leukocyte infiltration into the gastric mucosa (Bodger and Crabtree, 1998; Peek et al., 2010).

*H. pylori* virulence factors are the main cause of tissue damage, and include vacuolating cytotoxin (vacA), cytotoxin associated gene A (cagA), and neutrophil-activating protein (HP-NAP; Atherton, 1998; Cellini and Donelli, 2000; Fu, 2014). Binding of *VacA* to gastric cells triggers epithelial vacuolization and degeneration, loss of microvilli, and cytoplasmic disintegration (Goodwin et al., 1986; Papini et al., 1994; Garner and Cover, 1996; Smoot et al., 1996). Animal studies have demonstrated that purified *vacA* toxin causes gastric epithelial damage with little effect on inflammatory leukocyte infiltration (Telford et al., 1994; Ghiara et al., 1995). In addition, cagA has been shown to be strongly associated with development of local inflammation and expression of pro-inflammatory cytokines (Peek et al., 1995). Moreover, it has been suggested that the cagA gene and nearby sequences code for proteins that act synergistically and promote production and secretion of pro-inflammatory cytokines (Tummuru et al., 1995; Censini et al., 1996), and that the virulence factor HP-NAP promotes neutrophil adhesion, chemotaxis, and activation (Satin et al., 2000). The combined effects of these virulence factors is inflammation of local tissue caused by damage to gastric epithelial cells, and activation of pro-inflammatory cytokine production.

Histologically, infiltration of gastric tissue by leukocytes is a hallmark of *H. pylori* infection. In tissue biopsies collected from patients infected with *H. pylori* both neutrophil infiltration (Kamada et al., 2006; Jaramillo-Rodríguez et al., 2011; Xu et al., 2012) and increased infiltration of CD4+ T helper lymphocytes in the lamina propria (D'Elios et al., 1997a) have been reported. Further, in gastric mucosa, it has been demonstrated that *H. pylori* infection activates predominantly Th1-type immune responses (D'Elios et al., 1997a), and immunohistochemically analyses of gastric biopsies have revealed an increased presence of CD8+ lymphocytes and macrophages (Bedoya et al., 2003). Animal models established that the early stage of infection is marked by neutrophil infiltration (Rossi et al., 2000). As infection progresses, a drop in neutrophil counts is followed by increased tissue infiltration with mononuclear leukocytes, mostly CD3+ lymphocytes. Initially scattered, lymphocyte infiltrates organize into small patches in the corpus and antrum of stomach (Rossi et al., 2000; Sepulveda and Patil, 2008). Later, the appearance of CD4+ and CD8+ lymphocytes in the periglandular area and beneath the basal lamina correlates with histological signs of gastric epithelial damage. Eventually, leukocyte infiltrates became organized in follicles containing CD21+, CD4+, and CD3+ lymphocytes (Rossi et al., 2000). Furthermore, the appearance of neutrophils at late stage leukocyte infiltration suggests active chronic gastritis. Together, these data indicate that initially *H. pylori* infection causes neutrophil infiltration of gastric mucosa, then as infection progresses neutrophils are replaced by lymphocytes. This leukocyte infiltration is the main cause of gastric epithelial damage in *H. pylori* infected tissue.

Exposure of gastric epithelium to *H. pylori* results in the production of a number of cytokines that stimulate migration of immune effector cells into inflamed tissue, including upregulation of IL8, CCL5, CCL3, IFN, IL10, IL12p40, and IL18 (Crabtree et al., 1994, 1995a,b; Yamaoka et al., 1996, 1998; Karttunen et al., 1997; Park et al., 2001; Dzierzanowska-Fangrat et al., 2008). Studies using animal models have demonstrated that the early stage of *H. pylori* infection is characterized by increased expression of IL1, IL8, IL6, and TNF-α in gastric mucosa (Harris et al., 2000; Rossi et al., 2000). Then, as the disease progresses, IL8 expression declines, while IL1, IL6, and TNF-α remain elevated (Rossi et al., 2000; Harris et al., 2000). It has been suggested that at late stages of the disease, there is a shift toward Th1 immunity, involving cytokines such as IFNγ and IL12 (D'Elios et al., 1997a; Haeberle et al., 1997; Pellicanò et al., 2007), and that persistent activation of the Th1 immune response is a cause of tissue damage in *H. pylori* infection (D'Elios et al., 1997b; Smythies et al., 2000). A combination of transcriptional analysis of tissue biopsies and histological findings has provided most of the information about cytokine activation of *H. pylori* infection. However, there is limited knowledge of serum cytokine expression in children infected with *H. pylori*, since the majority of data is from adult populations (Bayraktaroğlu et al., 2004; Mehmet et al., 2004; Abdollahi et al., 2011; Eskandari-Nasab et al., 2013). To date, for the most part, cytokines studies have been limited to the Th1 or pro-inflammatory class. This is unfortunate, as *H. pylori* infection often occurs during early childhood establishing a lifelong chronic infection. Understanding cytokine expression at the initial stages of infection will identify early markers, and improve disease diagnosis.

Here, we present data on cytokine activation in serum of children with gastroduodenitis. Sixty-two juveniles with gastroduodenitis were included in this study, with 30 having a diagnosis of *H. pylori* infection. Regardless of the presence or absence of *H. pylori* pathogenicity markers, there was upregulation of the potent neutrophil chemoattractants, CXCL5, and CXCL6. However, serum levels of IL8, a prototype neutrophil attractant, were not statistically different between diagnoses. Therefore, our data suggest that a novel set of chemokines, CXCL5, and CXCL6, play a role in neutrophil trafficking into inflamed gastroduodenal tissue. Further, an intriguing observation was that the CCL25/GM-CSF ratio differed significantly between *H. pylori* positive and negative children with gastroduodenitis.

## Materials and methods

### Subjects

Sixty-two patients (24 boys, 38 girls; age 14.0 ± 2.1) hospitalized in the Children's Republican Clinical Hospital (Kazan, Russia) with a diagnosis of gastritis and duodenitis were recruited for this study. Initial diagnosis of *H. pylori* infection in 30 of the patients was based on clinical presentation and upper GI endoscopy. The presence of *H. pylori* was confirmed by urea breath test and PCR.

Biopsies were collected from each patient during upper GI tract endoscopy: two from antral part of the stomach along the major and minor curvatures, and 2–3 from the body of the stomach, along the major and minor curvatures. In addition, stomach biopsies were collected from three controls, who were found to be negative for any gastroduodenal pathology by diagnostic upper GI tract endoscopy. Biopsies were used for both PCR analyses and histological studies.

Serum samples were collected from all 62 juveniles with gastritis and duodenitis, as well as from 20 age- and sex-matched controls. All controls were negative for symptoms of upper GI tract infection or gastritis. Serum samples were stored at −80°C. The Ethics Committee of Kazan State Medical University approved this study (N6, 06.25.2012) and informed consent was obtained from the legal guardian of each study subject, in accordance with the Declaration of Helsinki and the article 20, Federal Law “Protection of Health Right of Citizens of Russian Federation” (N323-Φ3, 11.21.2011).

### Urea breath test

Breath ID Hp (Exalenz, USA) was used to confirm *H. pylori* infection. This breath test measures the presence of ^13^C labeled CO_2_ in the patient's breath after ingestion of a solution containing ^13^C labeled urea. After 10 min, exhaled air is collected and tested for the presence of ^13^CO_2_, which indicates *H. pylori* infection.

### PCR detection of *H. pylori*

DNA was extracted from biopsy tissue using the Helicopol Kit (Lytech, Moscow). *H. pylori* positive biopsies were analyzed by PCR for a pathogenicity marker profile using the Helicopol II Kit (Lytech, Moscow). Briefly, 2 μl total DNA was mixed with 4 μl 10x PCR buffer, 2 μl 25 mM MgCl_2_, 0.5 μl (100 pmol) each of primers, 40.7 μl distilled water, and 0.3 μl (2.5 U) Taq DNA polymerase. The reaction mixture was then subjected to 35 cycles, each consisting of 30 s at 94°C, 30 s at 50°C, and 2 min at 72°C. PCR products were analyzed on a 1% agarose gel.

### Cytokine analysis

Serum cytokine levels were analyzed using a Bio-Plex Pro Human Chemokine Panel (40-Plex; Bio-Rad, Hercules, CA), a multiplex magnetic bead-based antibody detection kit.

### Immunohistochemical analysis

After initial analysis, there were surplus clinical diagnostic biopsy specimens from 21 *H. pylori* positive and 13 negative cases. These were used for immmunohistochemical analysis. Punch biopsies were fixed in 4% paraformaldehyde for 4 h at 4°C, and then cryoprotected with 30% sucrose in PBS. Immunohistochemical staining was performed on 5 μm thick sections. Slides were deparaffinized with xylene and rehydrated through a graded alcohol series. Tissue morphology was evaluated by light microscopy using hematoxylin-eosin staining. Additionally, Alcian blue (pH 2.5) and periodic acid Schiff (PAS) staining were performed to detect the presence of sialomucins.

### Statistical analysis

Statistical analysis was performed using the STATISTICA 7.0 Software Package (StatSoft, Tulsa, OK) and the IBM SPSS Statistics 20 Software Package (IBM Corp, Armonk, NY, U.S.). Data are presented as the median (5–95% range) for continuous variables. Differences between independent study groups were tested by non-parametric methods. To address type 1 error, Kruskal–Wallis ANOVA by Ranks test for multiple independent samples was followed by the multiple comparison (all pairs) non-parametric *post-hoc* Steel-Dwass test Nonparametric multiple comparisons were made [recalculated and confirmed] by the Steel-Dwass all pairs test using JMP® 13.0.0 Software (SAS Institute Inc, USA). Differences were considered significant at *P* < 0.05. Jonckheere's non-parametric trend test was performed to compare three group medians when they were arranged in order. Cytokine profiles between subject groups were differentiated using forward stepwise discriminant function analysis.

## Results

### Patients

Sixty-two patients (24 boys and 38 girls) were admitted to the Children's Republican Clinical Hospital (Kazan, Russia) with a diagnosis of gastritis and duodenitis. The main symptoms were pain in the epigastric area, vomiting, nausea, headache, and fatigue. Biopsy samples collected from the antrum of the stomach were analyzed for the presence of *H. pylori* antigens. Thirty samples were positive for *H. pylori* and 32 samples negative. The surplus biopsy tissue from 21 positive and 13 negative juveniles (of the 62 enrolled in the study) was used for histological studies.

**Figure 1:**
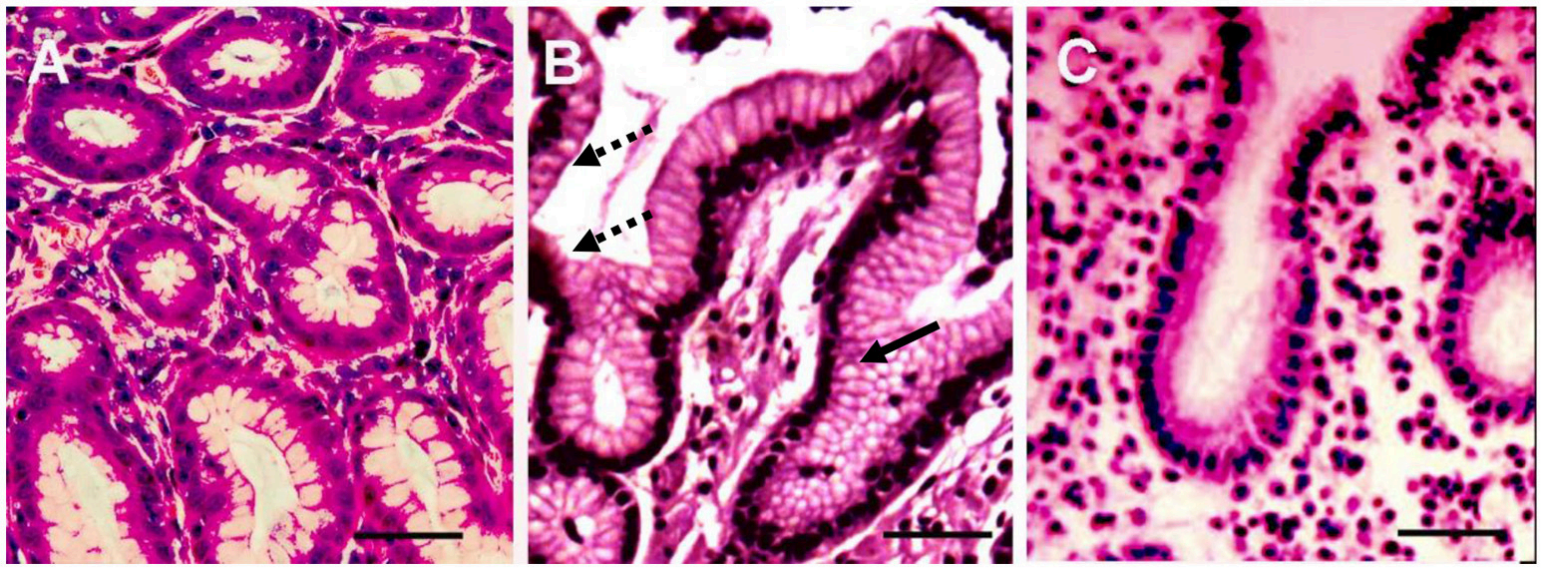
**Histological sections of three representative biopsies**. Gastric biopsy sections (3–5 μm) of control **(A)**, and *H. pylori* positive **(B)** and *H. pylori* negative **(C)** gastroduodenitis cases were deparaffinized and stained with hematoxylin and eosin (H&E). The gastric epithelium phenotype of the *H. pylori* positive juvenile **(B)** resembles the phenotype of colonic epithelium, characterized by multiple intracytoplasmic mucin droplets of varying sizes and shapes (solid arrow), and the absence of a brush border (dashed arrow). H&E; x100; Bar represents 20 μm.

**Figure 2:**
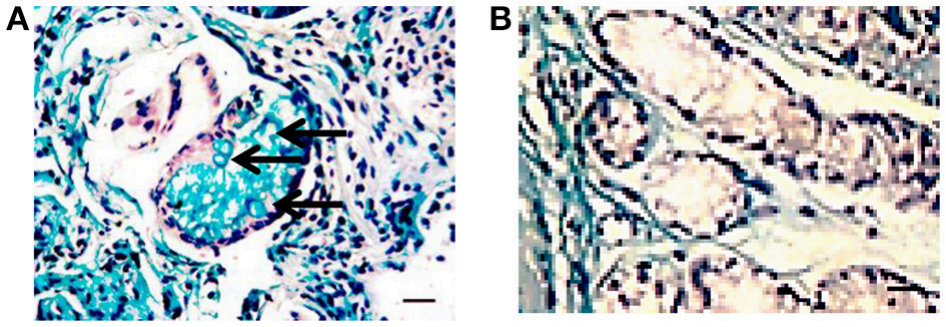
**Histochemistry of gastric epithelium metaplasia in a ***H. pylori*** positive biopsy**. Gastric biopsy sections (3–5 μm) were deparaffinized and stained with Alcian blue (pH 2.5) and PAS followed by H&E staining. Presence of sialomucins (stained blue, solid arrow) demonstrates incomplete metaplasia (Filipe et al., 1994). **(A)** *H. pylori* positive gastric biopsy; **(B)** *H. pylori* negative gastric biopsy (Bar represents 20 μm).

Three *H. pylori* positive biopsies had massive leukocyte infiltration of the lamina propria (Figure 1) with moderate incomplete metaplasia of the epithelium (Figure 2). Moderate lymphocyte infiltration of the lamina propria was found in 11 positive biopsies, with four having moderate epithelial metaplasia. Finally, four had moderate lymphocyte infiltration of the lamina propria with no sign of metaplasia. Based on histological evaluation, diagnosis of chronic atrophic gastritis was established in 9 (42.9%) of the *H. pylori* positive biopsies.

Histological analysis of the 13 *H. pylori* negative biopsies revealed epithelial metaplasia in eight biopsies, with lymphocyte infiltration in five cases. Two biopsies had lymphocyte infiltration and metaplasia. The remaining two biopsies had no sign of lymphocyte infiltration or metaplasia. Diagnosis of chronic atrophic gastritis was established in 6 (46.2%) of the *H. pylori* negative biopsies. The gastritis activity was graded in all biopsies according to the Histological Division of the Sydney System and “Up-Dated Sydney System” (Tytgat, 1991; Dixon et al., 1997; Table 1). This classification determines the activity grade based on the presence of polymorphonuclear leucocytes in combination with mononuclear inflammatory infiltrate, intestinal metaplasia, atrophy, and detection of *H. pylori* organisms. *H. pylori* positive biopsies were characterized by a higher grade of chronic inflammation compared to *H. pylori*-free biopsies (Table 1).

**Table 1:** **Histological examination of the gastric biopsies from ***H. pylori*** positive and negative gastroduodenitis cases**.

**Histological findings**	***H. pylori* positive, abs. (%), *n* = 21**	***H. pylori* negative, abs. (%), *n* = 13**	**Control abs. (%) *n* = 3**
No visible lymphocyte infiltration or few inflammatory cells	9 (42.9%)	12 (92.3%)	3 (100%)
Moderate leukocyte infiltration of the lamina propria (Grade I)	9 (42.9%)	1 (7.7%)	–
Severe leukocyte infiltration of the lamina propria (Grade II)	3 (14.2%)	0	–
Metaplasia (+)	12 (57.1%)	8 (61.5%)	–
Metaplasia (−)	9 (42.9%)	5 (38.5%)	3 (100%)

All patients were examined by gastroduodenoscopy. Hyperemia and edema were detected in the gastric mucosa of some, but not all, subjects. No visible changes were observed in the esophagus. Erosion with fibrin deposits, hyperemia, and edema were detected in 3 (14%) of *H. pylori* positive subjects. Five (16.1%) *H. pylori* positive cases were characterized by duodenal bulbar deformity with multiple scars, hyperemia and edema, with no deformation, or scarring observed in *H. pylori* negative stomachs. Two (6.4%) *H. pylori* positive stomachs had multiple ulcers, while all *H. pylori* negative patients were all ulcer-free. Overall, *H. pylori* positive stomachs were characterized by pronounced histological abnormalities compared to *H. pylori* negative stomachs.

### Cytokine profile

A total of 40 cytokines were analyzed in the serum of all subjects (Table 2). Children with gastroduodenitis had similar profiles, with the cytokine activation profile in *H. pylori* positive serum closely resembling *H. pylori* negative. For example, significant upregulation of CXCL2, CCL1, IL2, CCL7, CCL22, CXCL16, and CXCL12 was detected in both groups. Interestingly, the serum level of a major chemoattractant for neutrophils, IL8, did not differ significantly between patients with gastroduodenitis and controls. However, serum levels of CXCL5 and CXCL6, chemoattractant factors for neutrophils, were significantly upregulated in sera from all *H. pylori* positive juveniles, and not linked to the presence of neutrophil infiltration in biopsied tissue. Although serum levels of TNF-α were significantly higher in both *H. pylori* positive and negative sera compared to controls (Table 2), concentrations of this cytokine were within the normal range for this age (Mózes et al., 2011).

**Table 2:** **Serum cytokine profile of children diagnosed with ***H. pylori*** positive and negative gastroduodenitis**.

**Analyte**	***H. pylori* positive**	***H. pylori* negative**	**control**
	**Median (pg/ml; 5–95% range)**	**Median (pg/ml; 5–95% range)**	**Median (pg/ml; 5–95% range)**
IL-1β	11.10 (3.61–22.30); *P* < 0.05	8.93 (1.57–20.82); *P* < 0.05	2.50 (1.45–4.93)
IL2	38.24 (18.35–60.99); *P* < 0.05	32.13 (6.30–55.06)	17.98 (4.67–34.56)
IL4	64.28 (19.01–87.12); *P* < 0.05	58.32 (30.52–116.86); *P* < 0.05	8.40 (3.30–18.00)
IL6	28.30 (11.60–51.29)	25.84 (6.30–64.58)	16.22 (10.25–76.07)
IL8	29.00 (9.17–48.30)	25.59 (8.90–54.76)	8.32 (3.81–56.00)
IL10	142.41 (12.51–250.46)	119.02 (43.21–214.17)	77.95 (57.01–210.31)
IL16	2835.29 (91.60–11326.32); *P* < 0.05	2762.31 (381.46–6959.23); *P* < 0.05	678.23 (402.53–2713.07)
CCL1	203.26 (55.32–276.00); *P* < 0.05	178.84 (86.83–261.92); *P* < 0.05	46.00 (24.62–120.80)
CCL2	68.41 (3.23–164.97)	67.02 (12.29–100.79)	43.23 (34.30–345.63)
CCL3	24.12 (5.40–36.22)	21.51 (10.66–65.00)	28.99 (2.06–87.70)
CCL7	347.20 (21.11–595.61); *P* < 0.05	309.99 (95.47–550.32); *P* < 0.05	118.14 (61.00–448.84)
CCL8	87.86 (11.20–333.22)	115.22 (17.55–284.38)	78.49 (6.51–141.19)
CCL11	95.50 (47.20–156.00)	89.40 (63.60–135.80)	99.36 (57.30–167.20)
CCL13	210.14 (13.51–499.87); *P* < 0.05	251.50 (19.57–733.77); *P* < 0.05	34.27 (3.05–210.04)
CCL15	1654.79 (34.23–41566.08); *P* < 0.05	13334.57 (211.26–43075.98); *P* < 0.05	72.94 (23.65–242.02)
CCL17	518.65 (29.93–2962.22)	476.64 (95.91–1426.65)	243.07 (23.54–1341.02)
CCL19	1270.66 (47.13–4730.73)	1075.69 (194.00–2762.13)	642.28 (120.30–3283.56)
CCL20	35.41 (3.56–181.76); *P* < 0.05	45.20 (2.33–135.31); *P* < 0.05	4.34 (2.33–8.02)
CCL21	153.20 (134.20–195.40)	198.30 (156.20–225.30)	167.30 (136.20–201.20)
CCL22	3337.42 (116.17–8142.66); *P* < 0.05	3532.51 (515.56–5486.09); *P* < 0.05	378.38 (5.78–1858.06)
CCL23	570.75 (23.60–1450.51)	763.31 (46.20–1901.47)	252.75 (66.61–731.94)
CCL24	236.20 (156.50–328.80)	296.40 (178.30–301.69)	294.00 (166.00–315.00)
CCL25	1094.47 (60.90–2606.17)	351.70 (95.91–1426.65); *P* < 0.05	501.52 (29.40–2019.70)
CCL26	36.37 (26.20–78.67)	46.37 (29.30–66.29)	27.20 (21.27–78.40)
CCL27	96.67 (84.50–126.30)	113.34 (82.50–146.20)	85.60 (76.45–124.30)
CXCL1	877.52 (106.00–1495.04); *P* < 0.05	813.00 (340.69–2495.34); *P* < 0.05	327.00 (216.83–694.48)
CXCL2	924.71 (34.76–6357.27); *P* < 0.05	1506.60 (149.96–4479.89); *P* < 0.05	26.52 (5.36–55.10)
CXCL5	548.17 (126.00–2913.34); *P* < 0.05	758.31 (154.00–3379.31); *P* < 0.05	167.00 (123.00–209.00)
CXCL6	151.67 (34.00–479.22); *P* < 0.05	134.32 (64.54–711.35); *P* < 0.05	34.00 (34.00–108.23)
CXCL9	973.81 (58.21–2921.36)	876.86 (148.47–5175.09)	313.88 (114.60–1303.63)
CXCL10	568.92 (85.11–2082.65); *P* < 0.05	686.86 (89.18–3958.80); *P* < 0.05	87.00 (50.01–99.84)
CXCL11	135.81 (6.18–457.00); *P* < 0.05	126.97 (19.55–949.43); *P* = 0.000004	3.70 (1.78–12.26)
CXCL12	4423.42 (163.20–7225.97); *P* < 0.05	3659.66 (875.50–6827.47); *P* < 0.05	107.70 (89.40–1365.15)
CXCL16	1103.74 (9.53–2096.30); *P* < 0.05	1291.65 (128.47–1931.20); *P* < 0.05	171.11 (8.88–442.83)
CXCL13	86.49 (10.00–218.93)	97.56 (7.02–177.21)	11.05 (3.79–208.33)
CX3CL1	120.30 (67.10–176.50)	127.90 (66.80–150.30)	100.70 (68.30–167.20)
GM-CSF	8.30 (2.80–456.83)	33.59 (12.88–230.11); *P* < 0.05, ^*^P < 0.05	13.3 (10.20–15.30)
INF-γ	145.72 (15.26–243.31)	132.90 (36.60–245.16)	83.00 (40.25–164.07)
MIF	375.98 (78.00–46690.84); *P* < 0.05	6641.92 (525.81–89114.28); *P* < 0.05, ^*^P < 0.05	23.67 (9.28–207.56)
TNF-α	74.57 (2.56–131.86); *P* < 0.05	62.12 (24.66–104.25); *P* < 0.05	10.23 (3.45–36.97)

*P—significance between gastroduodenitis group and healthy control, Steel-Dwass test*.

* *P—significance gastroduodenitis groups, Steel-Dwass test*.

Significantly increased levels of CCL15, CCL20, and MIF were detected in both *H. pylori* positive and negative serum compared controls. However, only MIF levels differed significantly between the two groups. In contrast, no changes in serum levels of CCL2, CCL3, CCL8, CCL11, CCL17, CCL19, CCL21, CCL23, CCL24, CCL26, CCL27, CXCL9, CX3CL1, IL6, IL8, IL10, and IFN-γ were found either between the two groups of patients with gastroduodenitis, or between juveniles with gastroduodenitis and the control group.

We compared cytokine activation in *H. pylori* positive patients with different histological presentations. The histological data was used to divide the patients into four groups; group one was characterized by severe lymphocyte and neutrophil infiltration of the lamina propria with moderate epithelial metaplasia, group two had moderate lymphocyte infiltration of the lamina propria and moderate epithelial metaplasia, group three had mild infiltration of lamina propria and mild epithelial metaplasia, and group four had no lymphocyte infiltration and no epithelial metaplasia. The pattern of cytokine upregulation was similar in all groups and was characterized by increased serum levels of chemoattractants for lymphocytes, monocytes, natural killer cells and dendritic cells such as CCL1, CCL22, CCL15, CXCL16, and CXCL12.

Next, forward stepwise discriminant function analysis was utilized to identify cytokines that differed between control and *H. pylori* positive and negative sera. Fourteen cytokines were selected to generate a classification matrix [model summary: Wilks' lambda = 0.15498; *F*_(28, 102)_ = 5.6106, *p* < 0.0001, IL4, IL8, CCL3, CCL1, CCL7, IFNγ, CXCL12, CCL2, CXCL10, CCL23, MIF, TNF-α, CXCL1, and CXCL9]. Discriminant analysis revealed greater differences between patients with gastroduodenitis (both *H. pylori* positive and negative) and controls (Squared Mahalanobis distances 34.32) than between *H. pylori* positive and negative patients (Squared Mahalanobis distances 2.56). Furthermore, the 14 cytokine-based classification matrix yielded 100% correct predictions for controls (predicted classifications vs. observed classifications in the classification matrix), with lower percentages of correct predictions for cases that were *H. pylori* positive (80%) and *H. pylori* negative (76.66%).

**Figure 3:**
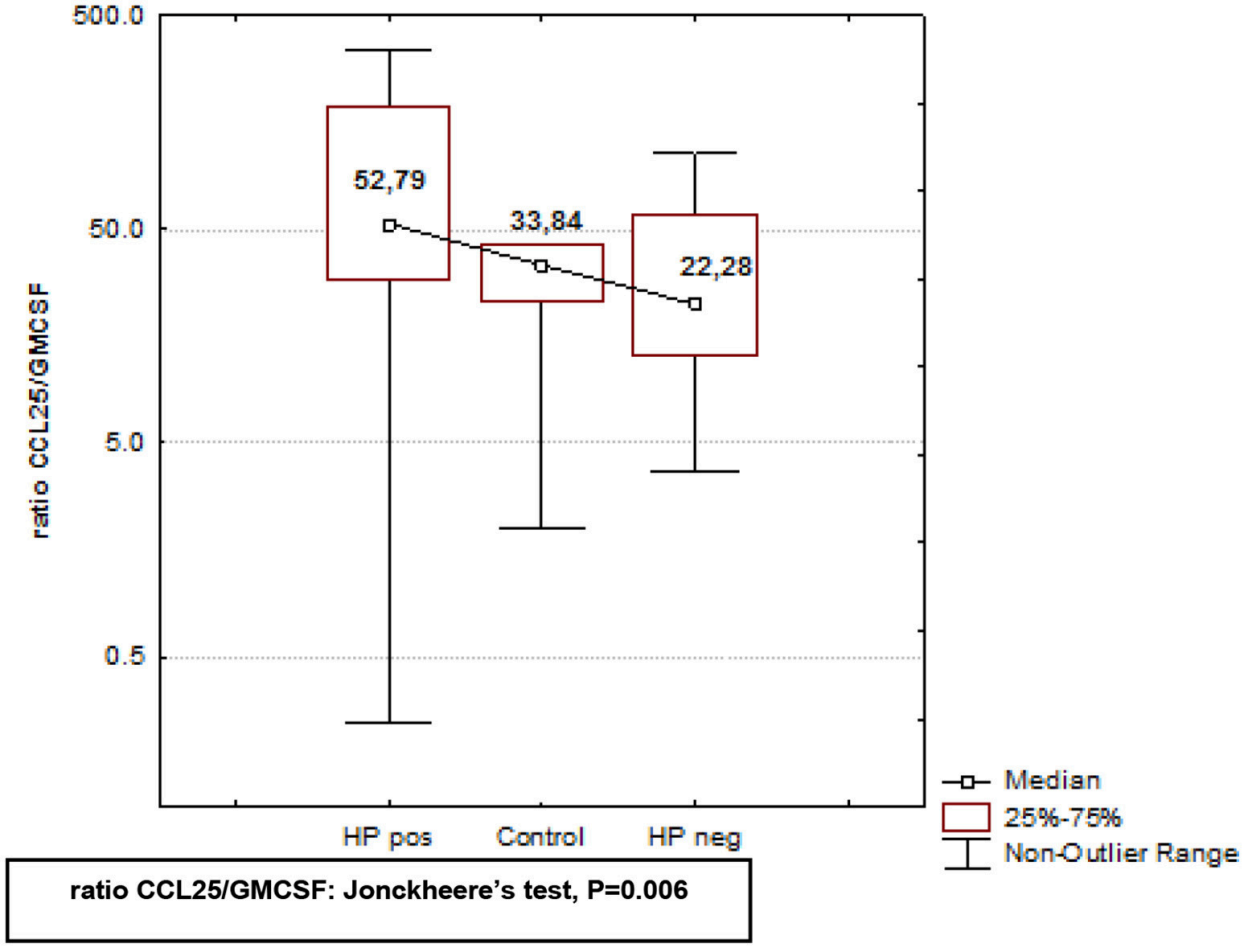
**Analysis of CCL25/GMCSF ratio in ***H. pylori*** positive and negative subjects, and healthy controls**. CCL25/GMCSF ratio in serum of *H. pylori* positive, *H. pylori* negative, and healthy controls was analyzed using using Kruskal–Wallis ANOVA by Ranks test, followed by the *post-hoc* non-parametric Jonckheere's-test for ordered medians. CCL25/GM-CSF ratio differed significantly between *H. pylori* positive and *H. pylori* negative subjects, and healthy controls (*P* = 0.006).

In Crohn's disease altered expression of GM-CSF and CCL25 has been suggested to play a role in the pathogenesis of inflammatory gastrointestinal disease (Samson et al., 2011). Therefore, we sought to determine whether these two cytokines were involved in the pathogenesis of *H. pylori*-related gastroduodenitis. Serum levels of GM-CSF were significantly upregulated in *H. pylori* negative subjects, while in *H. pylori* positive subjects, GM-CSF levels were similar to controls (Table 2), and significantly lower (^*^P < 0.05; Table 2) than *H. pylori*-free serum. In addition to increased GM-CSF levels, *H. pylori* negative serum was characterized by significantly lower levels of CCL25 (Table 2), suggesting bidirectional activation of these cytokines in negative serum. To further analyze the activation pattern of these cytokines in *H. pylori* negative and positive serum, we compared the CCL25/GM-CSF ratio in three independent study groups using Kruskal–Wallis ANOVA by Ranks test (*P*-level = 0.0083), followed by *post-hoc* Jonckheere's non-parametric trend test for multiple comparisons (*P*-level = 0.006; Figure 3). The CCL25/GM-CSF ratio differed significantly between each group. *H. pylori* positive and negative juveniles were positioned on either side of controls (Figure 3), suggesting that the CCL25/GM-CSFratio reflects an essential difference in gastroduodenitis pathogenesis with *H. pylori* positive and negative stomach having distinct forms.

## Discussion

*H. pylori* infection is often acquired early in childhood (McCallion et al., 1996; Suerbaum and Michetti, 2002; Tkachenko et al., 2007). This bacterium colonizes gastric mucosal epithelium, establishes a chronic infection (Marshall et al., 1985b; Morris and Nicholson, 1987), and once established, releases numerous virulence factors causing apoptosis and vacuolization of the gastric epithelium, and functional disruption of the gastric epithelial barrier (Goodwin et al., 1986; Papini et al., 1994; Garner and Cover, 1996; Smoot et al., 1996).

Currently, little is known about systemic activation of cytokines in children infected with *H. pylori*. The serum cytokine profile of infected children suggests a strong activation of chemoattractants for mononuclear leukocytes (Table 2). For example, in *H. pylori* positive gastroduodenitis we demonstrate increased serum levels of chemoattractants for mononuclear lymphocytes, such as CXCL10, CCL22, and CXCL16 (Taub et al., 1996; Andrew et al., 1998; Huang et al., 2008). This observation is supported by histological examination of biopsies, where increased leukocyte infiltration was detected (Table 1 and Figure 1). Interestingly, there were no differences in the serum cytokine profiles of patients with distinct histological presentations suggesting that tissue pathology is determined by *in situ* cytokine activation, which is not reflected in circulating cytokine levels. Serum levels of the prototype neutrophil chemoattractant IL8 (Gessler et al., 2004), remained unchanged in juveniles with gastroduodenitis. Bayraktaroglu et al described the same phenomenon in adults where IL-8 serum levels in *H. pylori* positive cases did not differ from controls (Bayraktaroğlu et al., 2004). However, increased levels of IL8 transcripts in tissue have been documented in *H. pylori* patients (Yamada et al., 2013; Nagashima et al., 2015), suggesting that upregulation of IL-8 may be a local characteristic of inflammation of gastrointestinal tissue. The molecular mechanisms regulating systemic neutrophil migration into inflamed tissue remains to be determined. Here, we demonstrate upregulation of CXCL5 and CXCL6, potent chemoattractants for neutrophils (Territo et al., 1989; Chertov et al., 1996; Mei et al., 2012) in serum from juveniles with gastroduodenitis. CXCL5, constitutively expressed by enterocytes, coordinates with CXCR2 the transmigration of neutrophils (Mei et al., 2012). Increased numbers of CXCL6 positive mucosal cells have been observed in Crohn's disease biopsies (Yamada et al., 2013), where, upon activation, upregulation of CXCL6 is more sustained than IL8 (Wuyts et al., 2003). It has been suggested that CXCL6 could play a role in supporting chronic inflammation by facilitating neutrophil migration at a late stage of infection (Wuyts et al., 2003). In this study, we present novel data on the upregulation of CXCL5 and CXL6 in serum during gastroduodenitis. Serum levels of these cytokines did not differ between *H. pylori* positive and negative children with gastroduodenitis, suggesting that upregulation of CXCL5 and CXCL6 is a tissue-specific response to inflammation, and not driven by a specific pathogen.

CXCL6 is a neutrophil chemoattracting factor produced by endothelial cells and macrophages exposed to IL1β or LPS (Wuyts et al., 2003). Additionally, transcriptional activation of both CXCL6 and CXCL5 has been demonstrated in cells stimulated with IL17 (Ruddy et al., 2004; Numasaki et al., 2005). Here, we show upregulation of IL17 in *H. pylori* positive juveniles suggesting that IL17 activation of epithelial cells and local macrophages causes upregulation of CXCL5 and CXCL6, which in turn promotes neutrophil chemotaxis into gastric tissue. It should be noted that CXCL6 synergy with MCP1 facilitates neutrophil chemotaxis (Gijsbers et al., 2005). Therefore, neutrophil chemotaxis may result from a combined action of several of cytokines.

The most intriguing observation made in this study was that GM-CSF and CCL25 may play a role in the pathogenesis of gastroduodenitis, and that changes in serum levels of GM-CSF and CCL25 may reflect the host's reaction to disease. Analysis of the CCL25/GM-CSF ratio indicates that *H. pylori* positive and negative gastroduodenitis are unrelated clinical entities. Our data support the observation made by Samson et al. suggesting a role for GM-CSF and CCL25 in the pathogenesis of inflammatory gastrointestinal disease (Samson et al., 2011). These authors demonstrated that CD patients with high serum level of GM-CSF neutralizing antibodies had increased number of iliac epithelial cells expressing CCL25. Local upregulation of CCL25 has been shown to facilitate iliac inflammation by stimulating CCR9-driven T lymphocyte migration. Our data provide further evidence for a role of both CCL25 and GM-CSF in the pathogenesis of inflammatory gastrointestinal diseases. Although our analysis revealed no correlation between the CCL25/GM-CSF ratio, histological presentation, and localization of gastrointestinal inflammation, we believe further study should be conducted to fully understand the function of CCL5 and GM-CSF in the pathogenesis of inflammatory gastrointestinal disease.

We have demonstrated upregulation of serum CXCL5 and CXCL6 in subjects diagnosed with gastroduodenitis, whereas serum levels of the neutrophil attractant, IL8, did not differ from controls. Therefore, we suggest that neutrophil accumulation in tissue in children with gastroduodenitis is directed by a distinct set of chemokines including CXCL5 and CXCL6. Additionally, we present the first evidence for a potential role for CCL25 and GM-CSF in the pathogenesis of gastroduodenitis, and that the CCL25/GM-CSF ratio can be utilized to for the discrimination of gastroduodenitis caused by *H. pylori* from gastroduodenitis due to other causes. Further study, using animal model for *H. pylori* gastroduodenitis is needed to determine the roles of CCL25 and GM-CSF in the pathogenesis of gastroduodenitis.

## Author contributions

SK, AR, VL, EM—cytokine profile analysis, SA, AK, DS, RF—clinical examination of patients, FK—morphology, YD and GC—biostatistics.

### Conflict of interest statement

The authors declare that the research was conducted in the absence of any commercial or financial relationships that could be construed as a potential conflict of interest.
